# Machine‐Learning Prediction of Metal–Organic Framework Guest Accessibility from Linker and Metal Chemistry

**DOI:** 10.1002/anie.202114573

**Published:** 2022-01-12

**Authors:** Rémi Pétuya, Samantha Durdy, Dmytro Antypov, Michael W. Gaultois, Neil G. Berry, George R. Darling, Alexandros P. Katsoulidis, Matthew S. Dyer, Matthew J. Rosseinsky

**Affiliations:** ^1^ Department of Chemistry and Materials Innovation Factory University of Liverpool 51 Oxford Street Liverpool L7 3NY UK; ^2^ Leverhulme Research Centre for Functional Materials Design University of Liverpool 51 Oxford Street Liverpool L7 3NY UK; ^3^ Department of Computer Science University of Liverpool Ashton Street Liverpool L69 3BX UK; ^4^ Present address: Nextmol (Bytelab Solutions SL) 08018 Barcelona Spain

**Keywords:** Database, Guest accessibility, Machine learning, Metal-organic frameworks, Porosity

## Abstract

The choice of metal and linker together define the structure and therefore the guest accessibility of a metal‐organic framework (MOF), but the large number of possible metal‐linker combinations makes the selection of components for synthesis challenging. We predict the guest accessibility of a MOF with 80.5 % certainty based solely on the identity of these two components as chosen by the experimentalist, by decomposing reported experimental three‐dimensional MOF structures in the Cambridge Structural Database into metal and linker and then learning the connection between the components’ chemistry and the MOF porosity. Pore dimensions of the guest‐accessible space are classified into four ranges with three sequential models. Both the dataset and the predictive models are available to download and offer simple guidance in prioritization of the choice of the components for exploratory MOF synthesis for separation and catalysis based on guest accessibility considerations.

## Graphical abstract

We present a machine‐learning tool that predicts the porosity of metal‐organic framework (MOF) materials based solely on chemical properties of their constituents: the metal and the inorganic linker. Our method leverages data available for thousands of synthesised MOF in prediction of properties of interest in potential synthetic targets without requiring knowledge of their structure but only requiring the knowledge of their constituents.

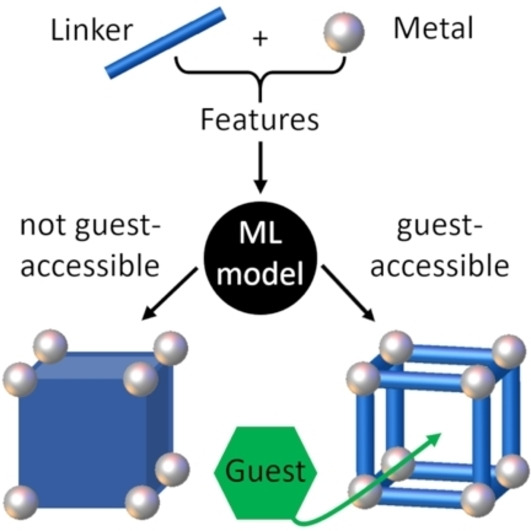

Metal–organic frameworks (MOF)[^1^, ^2^] are the focus of intense research interest because of their versatile potential for applications^[3]^ including gas sorption and separation,[^4^, ^5^] catalysis,^[6]^ and drug delivery.^[7]^ These hybrid solids, made by assembling inorganic centres and organic linkers, build on their reticular nature to offer a wide range of possibilities for the design of new materials with tailored chemistries and properties.^[8]^ Databases of hundreds of thousands of synthesized^[9]^ and hypothetical[^10^, ^11^, ^12^, ^13^] MOF structures are now available and used for computational screening^[14]^ in efforts focused largely on predicting gas sorption properties of a MOF with a given structure. The next step in maximizing the impact of these databases is to apply data science methods to the design of porous hybrid materials.^[15]^ Progress towards that goal has recently been thoroughly reviewed.^[15]^ In particular, a series of works[^16^, ^17^, ^18^] used MOF descriptors (some, such as pore sizes, require a priori knowledge of the MOF structure) to build a series of machine learning (ML) models for the prediction of CO_2_ and CH_4_ adsorption either from databases of hypothetical MOF structures[^10^, ^11^, ^12^, ^13^] or from the Computation‐Ready, Experimental (CoRE) MOF database of reported structures.^[19]^ However, databases of, or based on, existing structures only cover a limited part of the potential design space^[15]^ and new combinations of metal species and organic linkers are bound to lead to new MOF structures that arise from their coupled chemistries.

The objective of the present work is to harness the potential of ML to help chemists prioritise the available options from the earliest material design stage, at which only the chemical identities of the organic ligand and the metal species that are synthetically combined are known, in order to identify metal‐linker combinations with the highest likelihood of affording MOF structures that are accessible to guests. We address the specific case of three‐dimensionally connected MOF structures to ensure comparability of outcomes over lower‐dimensional counterparts such as coordination polymers with 2D and 1D networks of chemical bonds, though extension to these is straightforward. To achieve this objective, first, a dataset connecting 3D MOF structures to their chemical framework components, i.e., metals and linkers, was derived from the Cambridge Structural Database (CSD) MOF subset.^[9]^ Then, various ML models were evaluated to learn the connection between component chemistry and MOF properties without explicitly requiring a priori knowledge of the MOF structure. The most accurate of these models, a random forest classifier, predicts whether the structures produced by given metal‐linker combinations would be accessible to guests (i.e., adopt an open‐framework structure defined here as having a pore limiting diameter >2.4 Å) with 80.5 % accuracy, solely using the chemical descriptors of those metal‐linker combinations. This allows the researcher to assess the likely guest accessibility of a MOF based on the components without requiring knowledge of the structure, testing design hypotheses against the predictions of machine learning trained on all available experimental data.

The pore limiting diameter (PLD), i.e., the largest free sphere that can diffuse through the structure or equivalently the minimum restricting aperture along the diffusion path, is used throughout this paper to quantify MOF porosity while other properties of interest can be used instead to build similar models. To make our predictions for PLD more quantitative, we use a sequence of binary classifiers trained on different subsets of our data set to recognise the difference between small, medium and large pores as defined below. These predictive models, designed to guide the choice of MOF components for synthesis targeting separation and catalysis applications, together with the ML‐ready dataset of the constituents for 3D MOF reported in the CSD MOF subset, are available to download.

Recently, MOF deconstruction procedures were implemented to identify secondary building units (SBU) and linkers for computationally‐focused datasets.[^18^, ^21^] Here, we derived a dataset that connects the constituent linkers and metal atoms to the MOF structures directly from the reference repository of experimentally determined structures, namely the MOF subset of the CSD (Data Update 3‐2019), which contains more than 96 000 experimental MOF structures.^[9]^ The procedure illustrated in Figure 1 successfully decomposed 87.8 % (i.e., 28 994) of the identified 33 011 3D frameworks, while the labelling of the linker was ambiguous for the remaining 12.2 % of entries. The protocol successfully handles structures with disorder and is seamlessly transferable to 1D and 2D structures. Below we provide the summary of the protocol with full details available in the Supporting Information.

**Figure 1:**
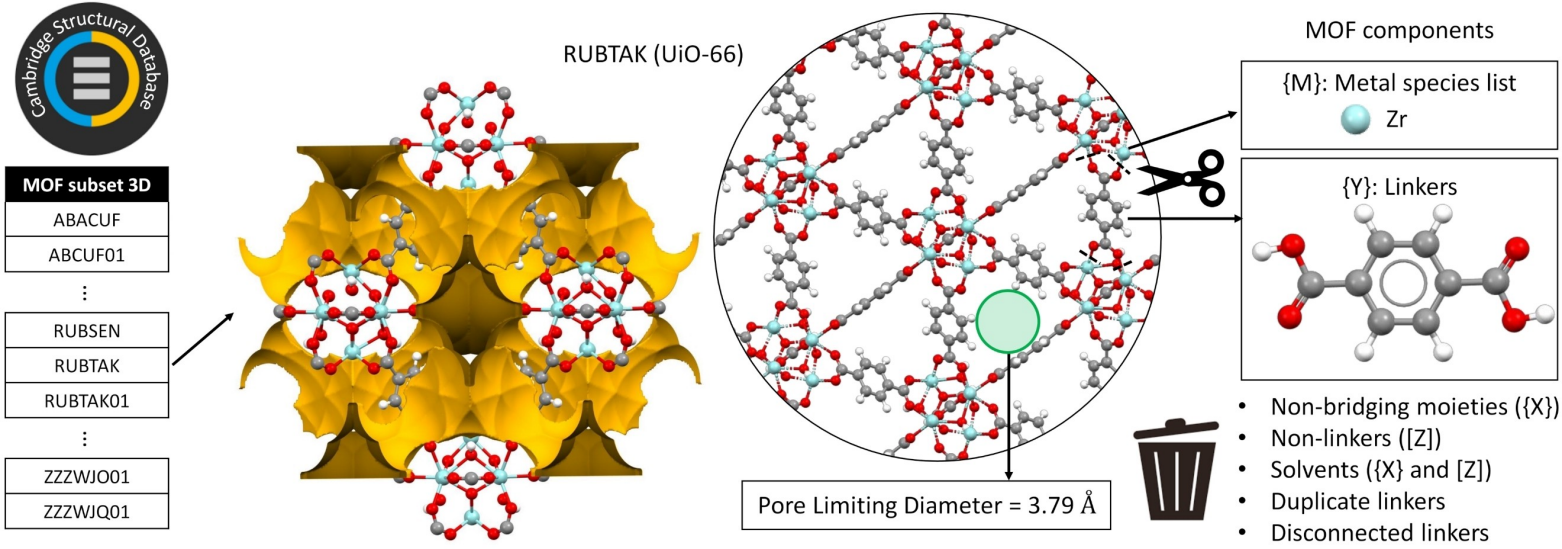
Classification of the 3D MOF component (organic linkers and metal species) dataset from the Cambridge Structural Database 3D MOF subset. Once the MOF structures are cleaned by removing species not bound to metal atoms, their porosity is evaluated by calculating their pore limiting diameter (PLD) with Zeo++. UiO‐66 (refcode RUBTAK)^[20]^ is shown here as an illustrative example and its Connolly surface, highlighting its porosity, is displayed for a probe diameter of 2.4 Å.

The formula unit of experimental MOF structures accessible via the CSD can be written as {M}{X}{Y} ⋅ [Z], where {M} is the list of metal atoms, {X} is the list of metal‐bound non‐bridging moieties, {Y} is the list of framework‐forming organic linkers and [Z] represent species, such as solvents or guests, that are located in the pores and are not bound to any metal atoms. After these non‐bonded species, [Z], are identified and removed using the CSD Python API,^[9]^ the resulting empty‐pore {M}{X}{Y} structures serve to define guest‐free structures. Their PLD is calculated with Zeo++,^[22]^ even though in some cases {X} includes solvent molecules coordinated to metal atoms that might be removed upon MOF activation. The retention of such species makes the estimate of porosity a lower bound but limits the risks of compromising framework integrity.^[9]^ Having removed all species [Z] not bonded to the MOF, still within the framework of the CSD Python API, a standard simplification algorithm^[23]^ is applied to separate the framework‐forming organic constituents {Y} from the metals {M} and non‐bridging moieties {X} bound to them, primarily cluster‐forming oxo and hydroxo species. Since the knowledge of moieties {X} is often not available before the MOF is synthesised, only the metal identity {M} and the linker identity {Y} will be used as the input for our predictive models.

To maximise the use of the data available in CSD, we adapted the decomposition procedure to deal with disordered MOF structures because they often contain at least one complete linker {Y} that can be recovered and included in the list (Figure S1). Once the structures of all physically sensible and unique linkers were identified, they were reduced to SMILES (Simplified Molecular Input Line Entry System)^[24]^ that we used to calculate 2D molecular descriptors and to generate representative 3D linker conformations using a series of Open Babel^[25]^ and RDKit^[26]^ scripts. This was done to avoid using any features of linker conformations extracted from CSD, as this information is not available without a priori knowledge of the MOF structure. This reflects the aim, which is to predict whether a MOF from a specific metal‐linker combination will be guest‐accessible without knowing which specific structure they will form: using information that is not available for the test set but is available for the structures that are used to train the model would constitute data leakage^[27]^ and is avoided by this protocol.

Following steps 1 and 2 in Figure 2, outlined above and depicted in Figure 1, we arrived at the database of metal and linker constituents for 28 994 3D MOF. 14 296 of these MOF had exactly one metal and a single linker and formed the dataset hereafter referred to as the “1M1L3D dataset” that was used to train our ML models. The other successfully decomposed MOF split over non‐mutually exclusive sets of 11 147 mixed‐linker and 5248 mixed‐metal structures (Table S1). Each entry in the 1M1L3D dataset (step 3 in Figure 2) contains metal identity, linker SMILES string, PLD and the corresponding CSD refcode. Using this dataset, we developed the ML approach (steps 4 to 6 in Figure 2) to predict MOF porosity from metal and linker chemistry. For researchers interested in separation and catalysis applications requiring guest access to the pores, this approach would allow the prioritization of metal‐linker combinations that have a higher likelihood of forming a guest‐accessible MOF in synthetic exploration.

**Figure 2:**
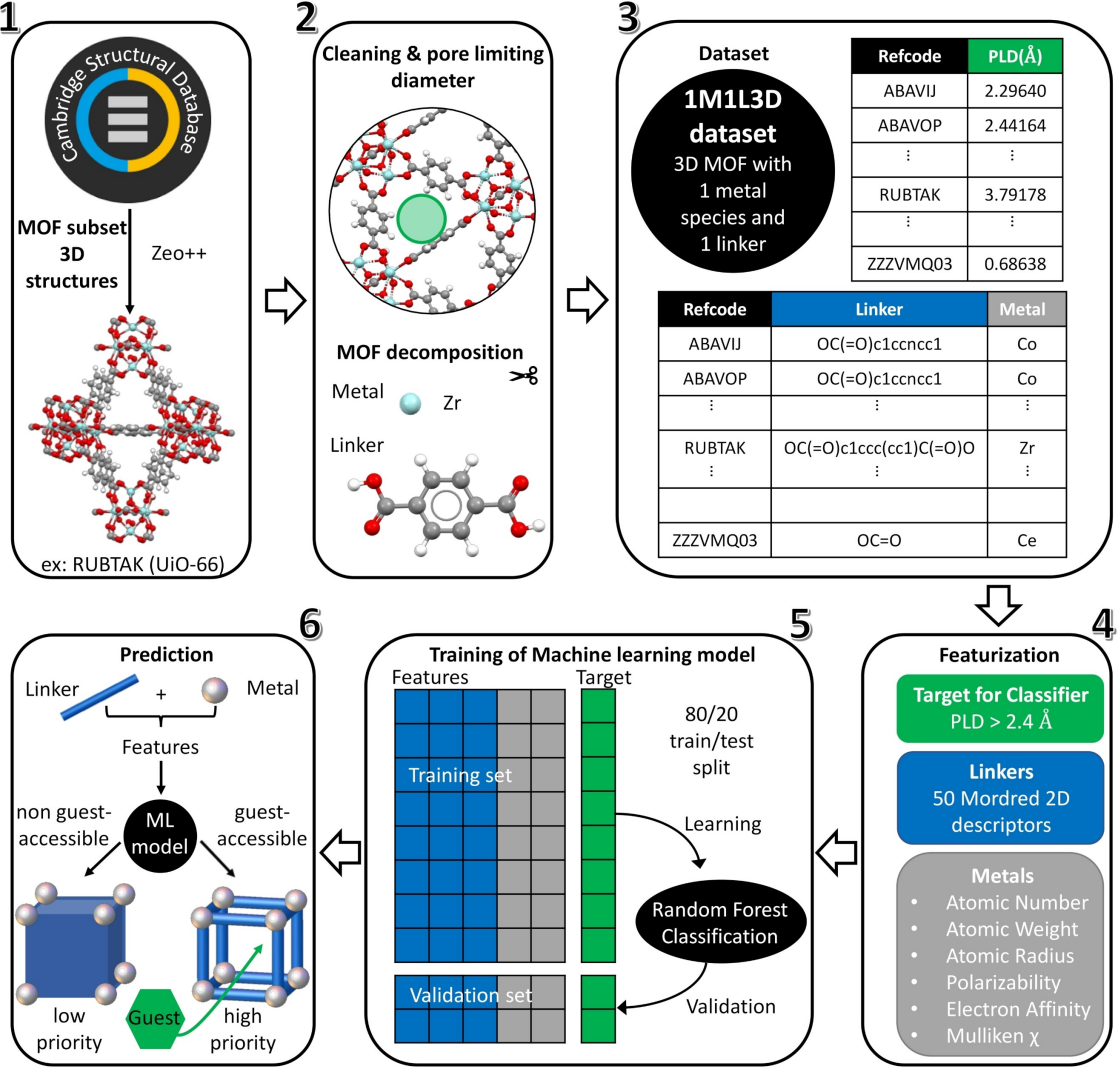
Workflow of creating the 1M1L3D dataset and using it to develop machine learning tools. The starting point is the information contained in the MOF subset of the experimental structures in the CSD that is used to select 3D MOF structures (step 1). These structures are decomposed into metal and linker (step 2) to produce the 1M1L3D dataset containing materials with a single metal and single linker (step 3). The evaluation of this dataset then takes place (step 4) to produce both the features (shown in blue for the linker and grey for the metal) and the porosity target (shown in green for one of the models as an example) on which the ML models are trained (step 5) to predict MOF guest accessibility (step 6). MOF are considered guest‐accessible when their pore limiting diameter is larger than 2.4 Å. The model is 80.5 % accurate in predicting guest accessibility based on the nature of the metal and the linker.

Following the convention adopted in the CoRE MOF database, we considered as porous the MOF structures with a PLD larger than 2.4 Å (approximately the van der Waals diameter of H_2_). Where several CSD structures corresponded to the same metal‐linker combination, we used the median PLD of these structures to represent the most likely outcome for the given metal‐linker pair (Figure S2).

The most accurate ML algorithm and feature set for our classification problem was identified through 3‐repeated stratified 10‐fold cross validation comparisons of nine classification algorithms (Figure S3), of five different feature sets each with different numbers of features (Figure S4), and manual adjustment of hyperparameters. The choice of learning algorithm weakly affected the accuracy here, as found in a study predicting shape persistence from a database of hypothetical porous cages.^[28]^ Testing details and hyperparameters used are available in Supporting Information. In summary, working with an 80/20 train/test split of the 1M1L3D dataset, a random forest classifier gave the highest accuracy (80.5 %) prediction of whether the combination of a given linker and a given metal would yield a guest‐accessible MOF of all the ML models evaluated. This random forest model, which we refer to as model 1, was trained on molecular descriptors from linkers SMILES codes, which rely only on two‐dimensional (2D) structural information, calculated via the free software Mordred,^[29]^ as linker features. The following six elemental descriptors were chosen as metal features: atomic number, atomic weight, atomic radius, polarizability, electron affinity and Mulliken electronegativity. Among the more than 1610 2D molecular descriptors provided by Mordred, the best performance (Table S2) was obtained with the set of 50 features selected via a SelectKBest procedure. All ML models were implemented using scikit‐learn library version 0.22.1.^[30]^

For some catalysis, separation and storage applications, beyond knowing whether or not a MOF will be accessible to guests, it is attractive to gain further insights on the pore size beyond the 2.4 Å cutoff in PLD dealt with by model 1, to be able to estimate whether a guest of interest can access the pores. With that objective in mind, we have analysed the distribution of PLD in the 1M1L3D dataset in order to determine two other PLD cutoff values, designed to maintain balanced datasets (Figure 3). Specifically, for the 7190 porous MOF with PLD>2.4 Å, by using a PLD cutoff of 4.4 Å we separate a subset of 3596 “small pores” MOF, i.e., 2.4 Å<PLD<4.4 Å, from a subset of 3594 MOF with PLD>4.4 Å. Additionally, a 5.9 Å cutoff splits this later subset between 1813 “medium pores” MOF, i.e., 4.4 Å<PLD<5.9 Å, and 1781 “large pores” MOF, i.e., PLD>5.9 Å. Using the parameters from the initial ML model, i.e., same random forest classifier with same hyperparameters and features, we adopted a sequential learning approach to train a second ML model (model 2) to predict whether a porous MOF would have a PLD between 2.4 Å and 4.4 Å, i.e., small pores, or a PLD larger than 4.4 Å. Then, we trained a third ML model (model 3) to predict whether a porous MOF with PLD>4.4 Å would have medium or large pores, i.e., respectively a PLD between 4.4 Å and 5.9 Å or a PLD>5.9 Å. These two ML models had accuracies of 76.4 % (model 2) and 68.5 % (model 3) since they were trained on respectively approximately a half (7190 MOF for model 2) and a quarter (3594 MOF for model 3) of the 1M1L3D dataset. The three sequential models allow us to quantify the PLD within predefined ranges and are provided ready for use in the evaluation of candidate metal‐linker pairs for new MOF synthesis.

**Figure 3:**
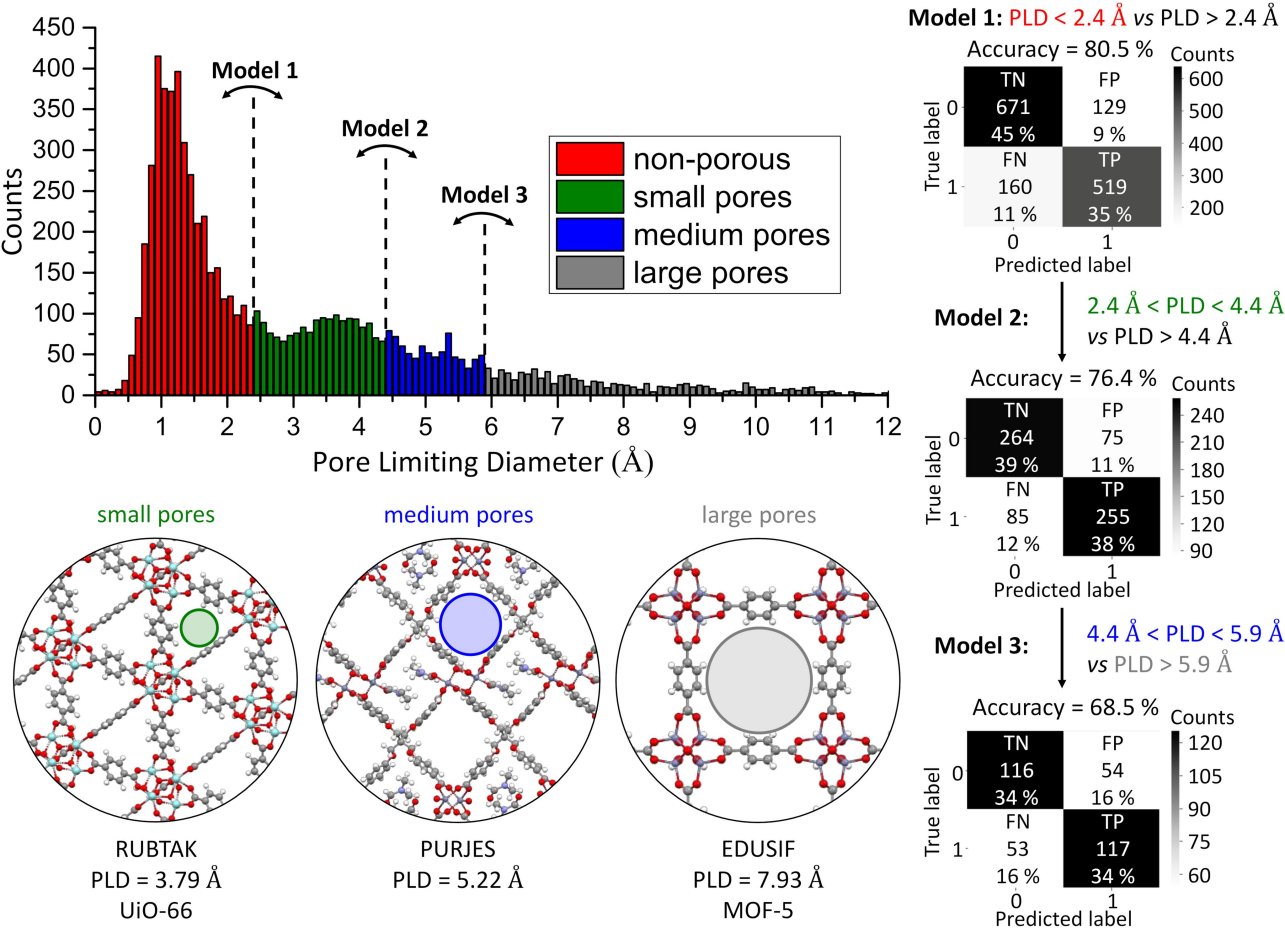
Sequence of three binary classifier models that predict the range of the pore limiting diameter (PLD) of a candidate MOF based on its linker and metal components. The four ranges are defined as non‐porous (PLD<2.4 Å, red), small pores (2.4 Å<PLD<4.4 Å, green), medium pores (4.4 Å<PLD<5.9 Å, blue) and large pores (5.9 Å<PLD, grey) and for each of the porosity ranges are illustrated by examples: UiO‐66 (refcode RUBTAK)^[20]^ for small pores, PURJES^[31]^ for medium pores and MOF‐5 (refcode EDUSIF)^[32]^ for large pores. The histogram shows the number of reported 3D MOF within each range for bin size 0.1 Å and finishes at 12.0 Å, which corresponds to 98.6 % of the 1M1L3D dataset, for clarity (otherwise the tail extends up to 71.51 Å). The confusion matrix for each model shows true negative (TN), true positive (TP), false negative (FN), false positive (FP) coloured according to their magnitude.

We have implemented a procedure that separates CSD‐deposited experimental MOF structures into metal and framework‐forming organic linker to produce a dataset that connects these synthetic constituents of MOF to the porosity of the reported experimental structures, specifically to their accessibility to guests. As an illustrative example, the 1M1L3D dataset is built from 3D‐connected MOF networks made of a single metal and a single linker species and used to train a random forest classifier that successfully predicts whether a MOF of this type, produced by a given metal‐linker combination, would be guest‐accessible, with an accuracy of 80.5 %. Two additional ML models are generated for use in sequence to predict whether the pores will be small, medium or large.

These ML approaches offer simple guidance to inform prioritisation of candidate metal‐linker combinations for synthetic exploration based on the likelihood of generating guest‐accessible MOF, and the match of potential pore dimensions to those required for sorption, separation and catalysis applications, with the aim of accelerating the discovery of open‐framework MOF structures beyond current structural databases.

## Conflict of interest

The authors declare no conflict of interest.

## Supporting information

As a service to our authors and readers, this journal provides supporting information supplied by the authors. Such materials are peer reviewed and may be re‐organized for online delivery, but are not copy‐edited or typeset. Technical support issues arising from supporting information (other than missing files) should be addressed to the authors.

Supporting Information

## Data Availability

The data that support the findings of this study are openly available in http://datacat.liverpool.ac.uk/1494.
