# Supplementary material for: Machine‐Learning Prediction of Metal–Organic Framework Guest Accessibility from Linker and Metal Chemistry
Source: Angew Chem Int Ed Engl. 2022 Jan 12;61(9):e202114573. doi: 10.1002/anie.202114573 (PMC9303542; doi:10.1002/anie.202114573)
Supplement: Supplementary file 1 — Supporting Information [file ANIE-61-0-s001.pdf]

Angewandte Chemie
Eine Zeitschrift der Gesellschaft Deutscher Chemiker

Supporting Information

Machine-Learning Prediction of Metal–Organic Framework Guest Accessibility from Linker and Metal Chemistry

*R. Pétuya, S. Durdy, D. Antypov, M. W. Gaultois, N. G. Berry, G. R. Darling, A. P. Katsoulidis, M. S. Dyer, M. J. Rosseinsky**

Table of Contents

Table of Contents....2

1 Decomposition of 3D MOF....3

1.1 3D MOF structures identification....3

1.2 MOF decomposition procedure....3

1.3 Decomposed dataset analytics....5

1.4 Multiple experimental structures in CSD produced by the same linker-metal combination....5

2 Machine learning procedures....6

2.1 Features....6

2.2 Algorithm performance....7

2.3 Final models....9

2.4 Using the Jupyter notebook....12

References....13

1 Decomposition of 3D MOF

1.1 3D MOF structures identification

The work presented in the main text specifically addresses the case of three-dimensional MOF structures – the structures in which, regardless of their porosity, the connectivity of the metal-linker network extends in all three dimensions. Therefore, the decomposition procedure reported in the next section has been applied to 3D frameworks. These MOFs were identified by combining the results from our structural analysis performed with Zeo++,[1] hereafter referred as Zeo-3D and the list of 3D MOFs curated and kindly provided by the Cambridge Crystallographic Data Centre (CCDC),[2] hereafter referred as CCDC-3D.

The reason behind using a combination of Zeo-3D and CCDC-3D lists of structures is that, while the version of the MOF subset the Cambridge Structural Database (CSD) used in this work (Data Update 3 – 2019) contains more than 96,000 entries,[3] the set of structures classified with respect to their dimensional connectivity provided by the CCDC totals 64,385 entries, covering mainly the entries from the so-called "Non-disordered MOF subset". The comparison of these two lists of 3D structures showed that about 94% of CCDC-3D MOF were also present in Zeo-3D, leaving 1,130 entries classified as 1D or 2D by Zeo++ (MOF dimensionality is defined by the dimensionality of the bonds network extension, namely 1 or 2 directions here). Among the structures in Zeo-3D MOF, only 228 were classified differently in the lists provided by the CCDC. To ensure that we did not leave out any 3D MOF structures for our study we considered as 3D all MOF present in either CCDC-3D or Zeo-3D sets.

1.2 MOF decomposition procedure

As already mentioned in the main text, the decomposition procedure implemented in this work to decompose MOF into their constituent components, *i.e.*, metals and linkers, deals with three-dimensional MOF, but it is important to highlight that it is seamlessly transferable to 1D and 2D structures. The formula unit of the experimental MOF structures accessible via the CSD can be written as {M}{X}{Y}·[Z], where {M} is the list of metal atoms, {X} is the list of metal-bound non-bridging moieties, {Y} is the list of framework-forming organic linkers and [Z] represent species, such as solvents or guests, that are located in the pores and are not bound to metal atoms.

While the identification of metals {M} is straightforward, the first step of the decomposition procedure, represented in the workflow Figure S1, is to identify and remove the non-bonded species [Z], present in the pores. This is done via a CSD Python API script,[3] that permits to output unique organic components (organic components with identical number of atoms and molecular weight are not output), both as SMILES codes and sdf structure files. Then, in-house scripts are used to remove residual bonded solvent molecules (CSD solvent list is used as reference) and non-linker species {X}, such as cluster-forming oxo- and hydroxo species. Because of the lack of standard SMILES definition,[4] both extracted SMILES from the CSD and canonical SMILES generated with Open Babel[5] were used. In order to generate neutral standardized canonical SMILES representation of the organic linkers, the extracted sdf files are processed using Open Babel.[5] Bond order information, not always reliable in the CSD, is deliberately eliminated via conversion to xyz format before generating new neutralized sdf files and its canonical SMILES. At each stage, disconnected linkers, identifiable by a dot in their SMILES code,[6] are usually the mark of some structural disorder and are thus removed, along with the duplicate linkers.

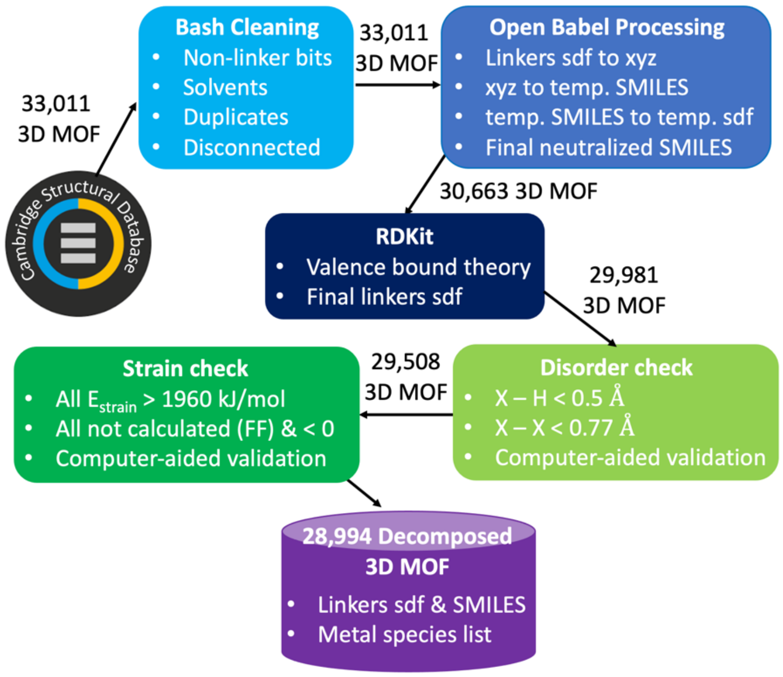

Figure S1. Steps of the procedure implemented to decomposed Cambridge Structural Database MOF's into their constituent components, i.e., metals and linkers. Next to the arrows are the numbers of CSD entries correctly processed at each stage of the decomposition procedure and passed on to the next stage.

Afterwards, the correctness of the produced neutral SMILES codes for the linkers, *i.e.,* their consistency with the valence bond theory, is checked with RDKit,[7] which is then used to generate the final 3D linker structures as sdf files. In that process, a 3D linker conformation for each linker is built from neutral SMILES and its geometry is optimized with the Merck Molecular Force Field (MMFF94).[8] Considering the amount of data, we could not assess every error or warning message, therefore any CSD entry generating warnings, for example in Open Babel processing or in RDKit kekulization, is discarded. After the bash cleaning and the processing of the linkers using Open Babel, the number of remaining entries is reduced by 7.1% from 33,011 initial 3D structures to 30,663 while RDKit checks discard an additional 2.0% leaving us with 29,981 entries.

Even with the precautions already taken, such as removal of disconnected linkers, valence bond theory checks with RDKit, removal of any linker structure that raises warning from Open Babel or RDKit, some corrupt linkers remain after the generation of the 3D molecular structures and are identified thanks to the procedure presented hereafter. One reason for this phenomenon is that the decomposition procedure was not restricted to non-disordered MOF structures, because disordered MOF structures often contain at least one complete linker that can be recovered despite the structural disorder. The trade-off being that this increases the chances of artificially generating corrupt linker structures from disordered linker conformations extracted from the CSD using the above workflow. Therefore, two additional steps of computer-aided validation are performed to identify the corrupted linkers originating from the disordered linker structures extracted from the CSD. These two steps, referred as disorder check and strain check, are the final steps of the workflow of Figure S1, and they are performed on the linker conformations, as extracted from the CSD. The linker structures that pass both checks and can be considered physically sensible, are reduced to SMILES and final representative 3D linker conformations are generated using a series of Open Babel and RDKit scripts. This was done to ensure that no feature of the linker confirmations extracted from the CSD is used in the machine learning procedure presented in the next section, as this information is not available without *a priori* knowledge of the MOF structure. Indeed, the aim of this work is to predict whether a MOF is guest-accessible solely on the basis of its metal-linker combination and therefore, using information that comes from the MOF structure to train the model would represent data leakage.

The first computer-aided validation step is a visual validation of all linker conformations containing particularly short interatomic distances (obtained via an in-house script). Disorder in the parent MOF structure, in particular when hydrogen atoms are missing, can lead to spurious linker structures unspotted by the valence bond theory check. Such linkers present unphysical atomic overlaps, characterized by interatomic distances shorter than 0.77 Å for non-hydrogen atoms and shorter than 0.50 Å when one of the atoms is hydrogen. The structures of these linkers are identified and collated together via in-house scripts to allow rapid and easy computer-aided validation of their disorder via visualization using the Jmol software. This set up allowed to flag 474 structures that were inspected manually and led to the discarding of 473, or 1.4% of the original set of 33,011 3D MOF structures. Because crystallographic structures in the CSD are often the average of various conformations in the crystals, it is frequent for the reported interatomic distances to be shorter that one would expect. Therefore, the cutoff distances used in that analysis to define what should be considered too short to be acceptable without risk are quite conservative but have been tuned in order to avoid visually inspecting an unnecessary large number of valid structures. It is therefore beneficial to add a second validation step which consists of visually validating the structures of the linkers exhibiting unexpected strain energies (very high, negative or structures yielding an error message), as defined by the energy difference between the fully relaxed neutral linker in the gas phase and its neutralized conformation extracted from the MOF, calculated with MMFF94 force field. Here again, the short interatomic distances in the CSD and the normal offset from the FF parametrization led to unreasonably high strain energy values. Therefore, a proper cutoff for over-strained structures requiring visual checking, while demanding a reasonable amount of human time, was found to be 1960 kJ/mol. For single linker MOF, a total of 450 entries were inspected resulting in 52 being discarded (43/350 at high strain and 9/100 in the low strain range) whereas for mixed-linker MOF 462 entries were discarded among the 867 inspected. In the case of mixed-linker MOF, when controlling the most strained entries, it is worth mentioning that while 216 of the 350 most strained entries were discarded only 27 of the next 117 (still higher than the 1960 kJ/mol threshold) followed the same fate. This highlights the adequate set up of the threshold value for visual inspection through which 1.6% of the total dataset is discarded. In total, during these two validation steps, 5.3% of the original dataset has been visually inspected which leads to the exclusion of 3.0%. Therefore, the remaining total number of successfully decomposed MOF is 28,994.

1.3 Decomposed dataset analytics

Here below, Table S1 presents the different types of 3D MOF successfully decomposed by the procedure implemented. The total of 28,994 MOFs were successfully decomposed into linker and metal components from the list of 33,011 3D MOFs and the number of unique linkers and metals was identified. Finally, 14,296 decomposed CSD 3D MOF structures made of a single metal and a single linker have been gathered in a dataset referred to as the “1M1L3D dataset”. This dataset, which connects CSD entries to MOF components, is used in the machine learning procedure presented below and is available to download.

Table S1. Different types of 3D MOF successfully decomposed.

Type of structures	Number of entries
Initial 3D MOF	33,001
3D MOF successfully decomposed	28,994
3D MOF with 1 linker	17,846
3D MOF with 2 linkers	10,632
3D MOF with 3 linkers or more	515
3D MOF with 1 metal element	23,745
3D MOF with 2 metal elements	4,894
3D MOF with 3 metals or more	354
3D MOF with 1 metal and 1 linker	14,296

1.4 Multiple experimental structures in CSD produced by the same linker-metal combination

Among the 14,296 entries of the 1M1L3D database, 7,391 metal-linker combinations are unique: 5,258 of them appear only once and correspond to a single entry in the CSD, 1,067 correspond to two CSD entries, 421 correspond to three CSD entries and the remaining 645 metal-linker combinations correspond to four or more CSD entries. There are multiple reasons why the same metal-linker combination can be reported in the CSD multiple times. The same MOF can be reported at different experimental conditions or/and with different solvents or guest molecules present in the pores. If such MOF is relatively rigid, its PLDs will be limited to a narrow range and will only be weakly affected by the experimental conditions and the presence of different guests in the pores (for example, 3.79 Å < PLD < 3.99 Å for UiO-66 structures) but if such a MOF is flexible, its PLD can cover a relatively large range (for example, 2.51 Å < PLD < 7.21 Å for MIL-47(Al) structures). In addition, MOFs can have detachable ligands that partially block the pores when they are coordinated to the metal (for example, water in HKUST-1 MOF) or have different topologies produced by the same metal-linker combination (for example, HKUST-1 and COK-18 both made from Cu-BTC). Figure S2 demonstrates the range of PLDs observed across the 86 CSD entries that all correspond to copper metal and benzene-1,3,5-tricarboxylate (BTC) linker combination. These are mainly multiple entries of hydrated and dehydrated HKUST-1 but other porous and non-porous materials that utilise this metal-linker combination are also present (Fig. S2).

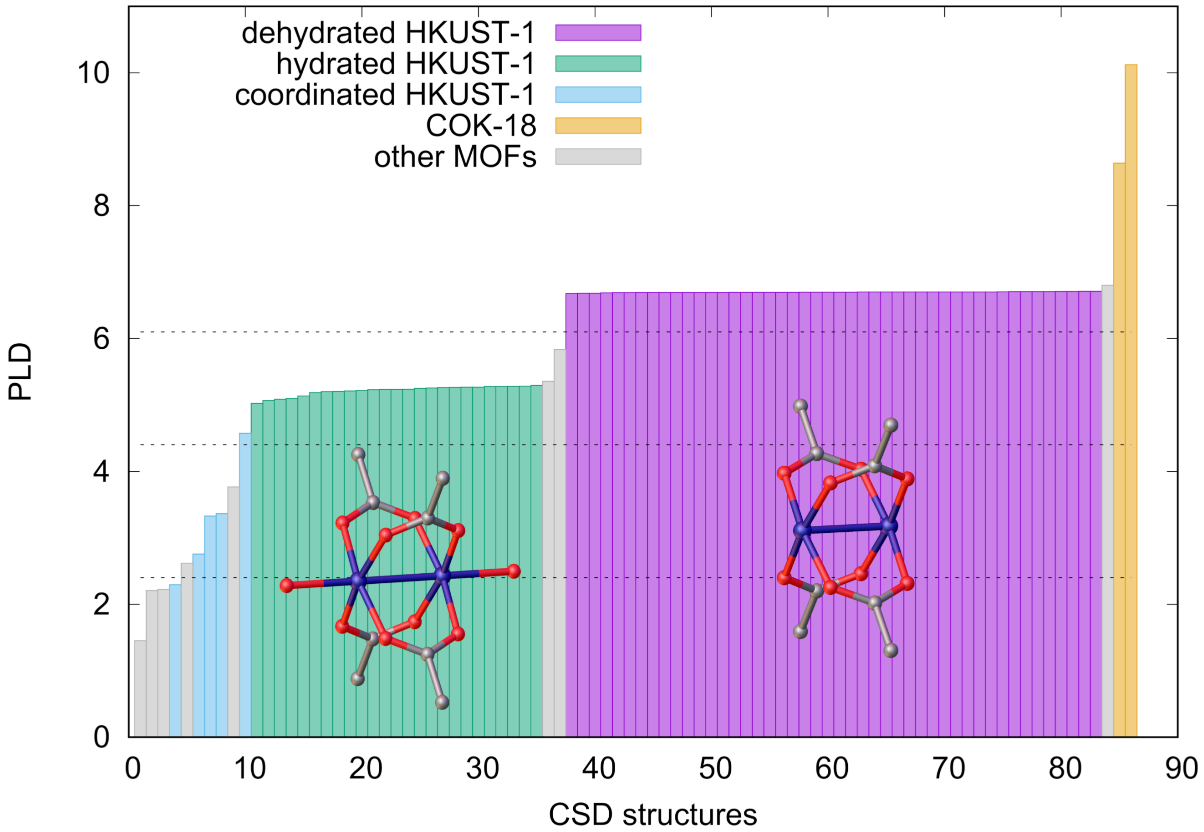

Figure S2. The distribution of pore limiting diameter for the 86 CSD entries that are all produced by the same copper metal and benzene-1,3,5-tricarboxylate (BTC) linker combination coloured by the MOF structure they correspond to. The majority of these CSD entries correspond to dehydrated HKUST-1 (purple). When water molecules (green) or other monodentate ligands (blue) are coordinated to the copper paddlewheel cluster, the pores are partially obstructed and the PLD is reduced compared to that of the dehydrated HKUST-1. The median PLD value of 6.69 Å was used for training the ML model for Cu-BTC metal-linker combination.

The majority (651 or 61%) of the 1067 metal-linker combinations in the 1M1L3D dataset that corresponded to exactly two entries in the CSD, had a difference in their PLDs of less than 0.5 Å, and 779 or 73% had a difference in their PLDs of less than 1.0 Å. This meant that only in 171 or 16% of these pairs was the smaller PLD below 2.4 Å, while the larger PLD was above 2.4 Å. Therefore, there was an ambiguity about whether this metal-linker combination produces a MOF whose pores are guest-accessible or not guest-accessible. For the remaining 896 or 84% of these pairs there was no ambiguity as the corresponding CSD structures fell into the same class – 480 had both PLDs below 2.4 Å, and 416 had both PLDs above 2.4 Å.

Similarly, for the 421 metal-linker combinations in 1M1L3D dataset that corresponded to exactly three entries in the CSD, the difference between the smallest and the largest PLD was less than 1.0 Å in 204 or 48% cases. This meant that in 265 (63%) out of 421 cases assigning the porosity label to the metal-linker combination was unambiguous as the three CSD structures had their PLDs either all below 2.4 Å or all above 2.4 Å.

Overall, for the 7,391 unique metal-linker combinations in 1M1L3D dataset, when considering the ambiguity in PLD being greater or less than 2.4 Å, the porosity label was unambiguously assigned in 6738 or 91% cases with this number slightly decreasing to 6480 or 88% cases when considering the ambiguity of the PLD belonging to one of the four predefined ranges considered in this paper.

Where the labelling was ambiguous due to the range of reported PLDs (as in the example of Cu+BTC discussed above), we used the median PLD value as the representative experimental outcome for this metal-linker combination. The analysis above indicates that only about 10% of the metal-linker combinations were affected.

2 Machine learning procedures

2.1 Features

In this study several different sets of features have been tested and are presented in this section. First, a simple encoding for the chemical composition of the components was considered. Then, the chemical descriptors for both linker and metal components were used. Specifically, we explored the effect of using only molecular descriptors calculated from SMILES strings (2D descriptors) and supplementing them with the descriptors calculated from 3D linker conformations using two software packages.

As the 1M1L3D dataset contains the linker and metal of the 3D MOF made of a single metal and a single linker, it is straightforward to obtain the chemical composition of each entry. In order to maintain a fixed length vector for all the entries in the dataset, all chemical elements encountered throughout the entire dataset are accounted for in each entry: either by their number of atoms in each linker or by a 0 if the element is not present. Similarly, for the metal, the metal encountered in the given entry is marked as 1 whereas all other

metals are marked as 0. The total length of this vector encoding composition is 70, which corresponds to the total number of distinct chemical elements encountered throughout the 1M1L3D dataset (17 distinct chemical elements for the linkers and 53 distinct metals).

To improve the accuracy of the predictions, we also trained ML models using molecular descriptors as linkers' features. Molecular descriptors from two packages have been compared, namely Dragon6,[9] a commercial package, and Mordred,[10] a freely available package. With both packages, molecular descriptors relying on the 2D and on the 3D molecular structures of the linkers can be calculated. The packages are able to access the 2D structures from the linkers SMILES codes provided as input, but it is necessary to explicitly provide 3D molecular structures generated beforehand to calculate 3D descriptors. It is worth mentioning that, unlike Mordred, Dragon 6 is able to calculate 3D descriptors that use atomic partial charges. The partial charges for the linkers were assigned using Open Babel[11] implementation of Gasteiger-Marsili sigma partial charges.[12] With Mordred we have calculated 1,613 descriptors from the linkers' SMILES and 1,826 from linkers' 3D molecular conformations. Respectively, Dragon6 returned 2,098 descriptors from the SMILES (the low variance descriptors, threshold = 0.0001, were already filtered out) and 3,582 descriptors from the 3D molecular structures. The effect of using either only 2D descriptors or both 2D and 3D descriptors has been tested and is reported in the next section.

To describe the metal when molecular descriptors were used as linker features, the following six elemental descriptors were chosen as metal features: atomic number, atomic weight, atomic radius, polarizability, electron affinity and Mulliken's electronegativity.

For all ML models, the features used in this study were preprocessed similarly, first by applying the standard scaler procedure implemented in scikit-learn,[13] and then by discarding all nearly-constant features with variance below 0.0001.

2.2 Algorithm performance

In order to identify the most suitable learning algorithm, a number of classification algorithms were tested on 2D descriptors calculated with Mordred to predict whether a MOF is guest-accessible or not, *i.e.,* has a pore limiting diameter (PLD) larger than 2.4 Å. All ML algorithms were implemented in Python 3.6 using the versatile scikit-learn library version 0.22.1[13] and their performance was compared via a 3-repeated stratified 10-fold cross validation procedure. The performance of each algorithm depends on the number of features used to train the models, therefore the classification algorithms listed hereafter have been tested with a series of different number of features selected via the SelectKBest procedure:

- logistic regression (LR): with a 'liblinear' as optimization algorithm (solver='liblinear') to perform (multi_class='ovr').
- linear discriminant analysis (LDA): with default parameters.
- k-nearest neighbors (KNN): with default parameters.
- decision tree classifier (CART): with default parameters.
- Gaussian naive Bayes (NB): with default parameters.
- Support Vector Machine (SVM): with default parameters.
- bagging classifier (BC): with default parameters.
- random forest classifier (RF): with default parameters.
- multi-layer perceptron classifier (NN_MLP): with a maximum iteration number increased to 500 (max_iter=500). By default, this is configured to have one hidden layer of size 100, and ReLU activation functions, optimised by the Adam gradient descent algorithm

The relative performance of different classification algorithms was assessed by their accuracy, which is the ratio between the number of correct predictions and the total number of input samples. Figure S3 shows that, as we increased the number of features from 10 to 1000, the random forest classifier (RF) algorithm consistently yields the best accuracy over the entire range of numbers of features. Therefore, the RF was chosen to train the models tested hereafter and those reported in the main text.

SUPPORTING INFORMATION

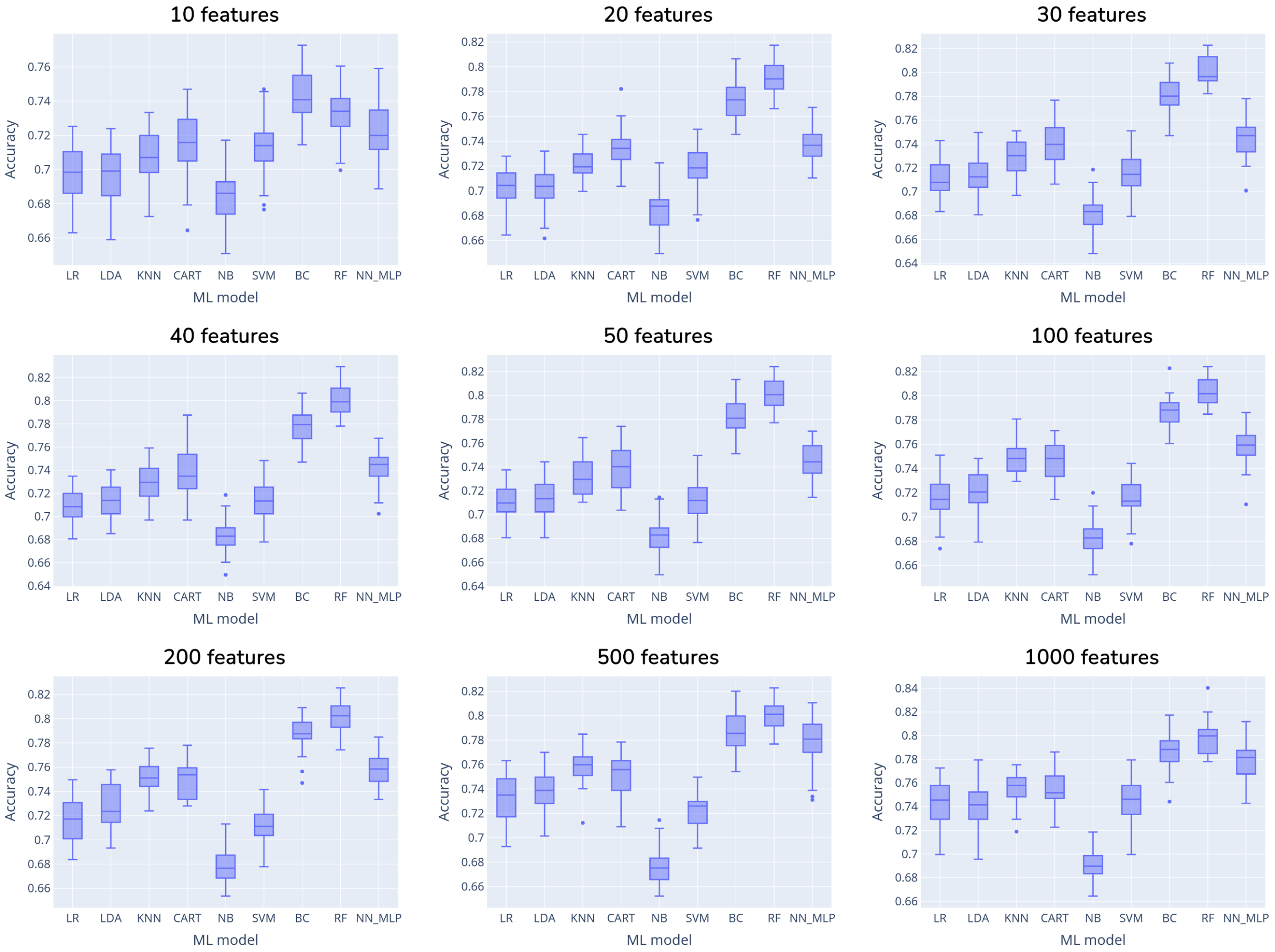

Figure S3. Box plot representations of ML algorithms' accuracy when trained with different numbers of features. The boxes account for the 25th to the 75th percentile and the horizontal line inside each box represents the median value of the results. The horizontal lines outside the boxes represent the results within 1.5 interquartile ranges of the lower and upper quartiles. The results that lie outside the boxes and lines areas are represented as points.

Once the RF algorithm was selected as the best approach, the performance of RF models was evaluated for five different sets of features presented in the previous section:

- composition features only
- 2D molecular descriptors (calculated with either Mordred or Dragon 6)
- 2D and 3D descriptors (calculated with either Mordred or Dragon 6)

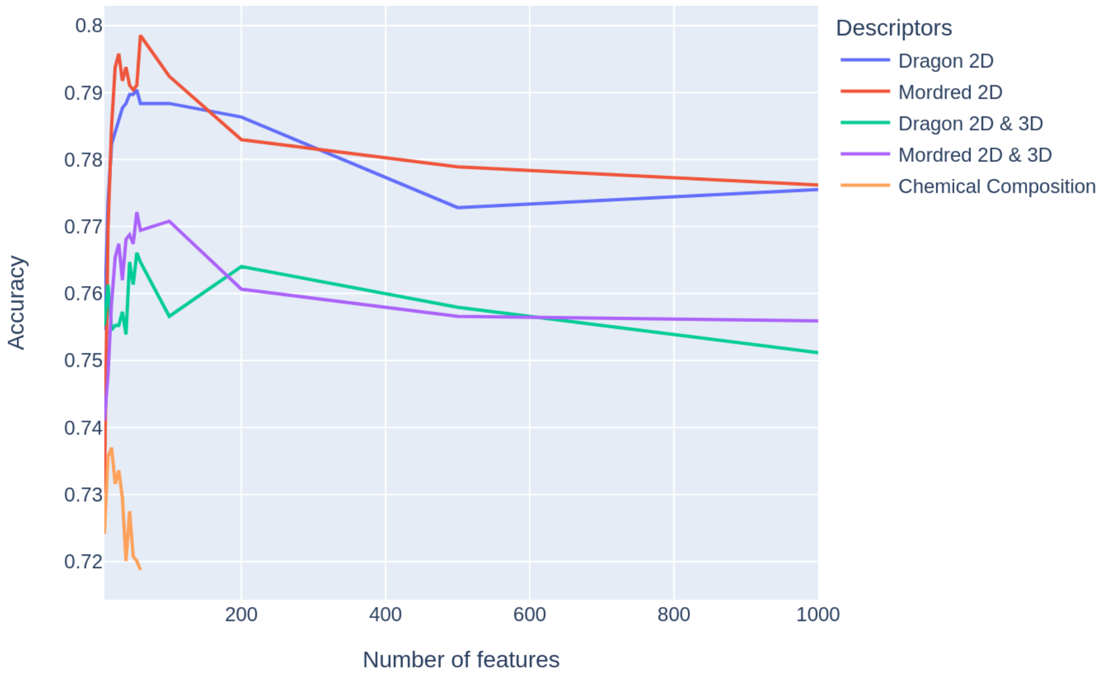

Figure S4. Accuracy of the random forest models trained with different sets of features as a function of the number of features.

The same 3-repeated stratified 10-fold cross validation procedure was employed for each set of features. Different numbers of features were tested in order to identify the combinations yielding the best performance. In all cases, we found that peak performance was observed when 30 to 50 features were used, while adding additional features did not improve accuracy of the models. Figure S4 shows how accuracy changes as a function of the number of features for each set of features and we have decided to use 50 features to describe the linker in the final models presented in the next section. According to Figure S4, models simply trained on chemical composition had the lowest accuracy but still performed surprisingly well. The RF algorithms trained on 2D molecular descriptors, either from Mordred or Dragon 6, consistently performed best over the entire range of numbers of features compared to other approaches. It is interesting to note that the addition of the 3D descriptors, either from Mordred or from Dragon 6, reduced accuracy by approximately 2.5%. The results from models trained on the 2D molecular descriptors from either Mordred or Dragon 6 are close to each other. Therefore, since the Mordred package is also freely available, we have chosen to use 50 of its 2D descriptors to build the final models presented in this work to allow access to our models free from any licensing issues.

2.3 Final models

Once the ML algorithm and the set of features were chosen, the hyperparameters of the RF algorithm were investigated manually. We found that the RF algorithm performs already quite well with the default settings and the changes of the hyperparameters could produce only a slight increase in performance. It was therefore decided that the sequence of three models developed in this paper to classify the range of the 3D MOF guest accessibility would be trained on the 50 Mordred 2D molecular descriptors previously identified and would rely on RF algorithm with:

- 2000 trees in the forest (chosen to balance performance with time complexity)
- at least 2 samples to split a node
- at least 1 sample per leaf
- a minimum weighted fraction per leaf of 0.0
- a maximum number of features considered per node split of log2
- an unlimited number of leaves per node

In addition to the confusion matrix reported in the main text in Fig. S4, in Tab. S2 we report various performance metrics for the final three RF models.

Table S2. Performance metrics of the three sequential classification models.

Performance metric	Model 1	Model 2	Model 3
Accuracy	0.805	0.764	0.685
Balanced accuracy	0.802	0.764	0.685
Precision	0.801	0.780	0.689

Recall	0.764	0.750	0.676
F1 score	0.782	0.528	0.371
Matthews correlation coefficient	0.605	0.528	0.371
Cohen's kappa	0.605	0.528	0.371
Jaccard score	0.642	0.614	0.522
Zero one loss	0.195	0.235	0.315
Hamming loss	0.195	0.235	0.315

As described in the main text, the 1L1M3D dataset is well-balanced as approximately half of its distinct entries have the pore limiting diameter PLD > 2.4 Å and are classified as guest-accessible. The cut-off values of PLDs for models 2 and 3 were chosen so the resulting subsets of 1L1M3D are also well-balanced. This explains why the accuracy is similar to the balanced accuracy. Both accuracy and balanced accuracy decrease from model 1 to model 3 as the size of the training set roughly halves as we go from model 1 to model 2 and then halves again as we go from model 2 to model 3.

As expected for binary classification where the true positive rate is approximately equal to the true negative rate, the precision, recall and F1 score have similar values to accuracy. This is true for all three models. Similarly, there is little difference between Matthews coefficient and Cohen's kappa. The zero one loss and Hamming loss are practically identical to each other for each model and increase from model 1 to model 3 due to the reduction in the training set size.

To quantify uncertainties, the three RF classifiers were evaluated through a 3-repeated stratified 10-fold cross validation procedure. The results are summarized in Table S3.

Table S3. Performance metrics of the three sequential classification models in the 3-repeated stratified 10-fold cross validation procedure. The average values are reported together with their standard deviations.

Performance metric	Model 1	Model 2	Model 3
Accuracy	0.752 ± 0.018	0.692 ± 0.029	0.630 ± 0.028
Balanced accuracy	0.747 ± 0.018	0.692 ± 0.029	0.629 ± 0.028
Precision	0.739 ± 0.026	0.691 ± 0.032	0.635 ± 0.032
Recall	0.698 ± 0.027	0.691 ± 0.050	0.651 ± 0.041
F1 score	0.717 ± 0.021	0.690 ± 0.033	0.642 ± 0.027
Matthews correlation coefficient	0.498 ± 0.037	0.385 ± 0.058	0.259 ± 0.056
Cohen's kappa	0.497 ± 0.036	0.383 ± 0.058	0.258 ± 0.056
Jaccard score	0.560 ± 0.025	0.528 ± 0.038	0.473 ± 0.023
Zero one loss	0.248 ± 0.018	0.308 ± 0.029	0.370 ± 0.028
Hamming loss	0.248 ± 0.018	0.308 ± 0.029	0.370 ± 0.028

As mentioned earlier, the final RF models were trained using the same set of features, namely 50 2D molecular descriptors calculated with Mordred for the linkers and 6 elemental descriptors for the metal species. The complete list of features is listed hereafter, sorted according to their importance in the training of model 1, given in parentheses.

1. feature Atomic Radius (0.043653)

2. feature polarizability(A^3) (0.042725)
3. feature Mulliken EN (0.041033)
4. feature Atomic_Weight (0.037770)
5. feature Atomic_Number (0.037709)
6. feature electron affinity(kJ/mol) (0.030257)
7. feature ETA_eta_FL (0.026788)
8. feature VR2_Dt (0.026359)
9. feature VR2_DzZ (0.026318)
10. feature VR2_Dzm (0.025383)
11. feature MID_C (0.024599)
12. feature ATS1dv (0.024351)
13. feature Xp-3d (0.024132)
14. feature BertzCT (0.022889)
15. feature SpAD_A (0.022759)
16. feature SpAbs_A (0.022261)
17. feature VR2_Dzp (0.022180)
18. feature VR2_Dzse (0.021796)
19. feature VR2_Dzpe (0.021382)
20. feature VR2_Dzare (0.021352)
21. feature ETA_beta (0.021216)
22. feature TpiPC10 (0.021106)
23. feature VR2_Dzi (0.020561)
24. feature VR2_Dzv (0.020347)
25. feature PEOE_VSA7 (0.019510)
26. feature VR2_D (0.018618)
27. feature Xp-7d (0.018500)
28. feature SMR_VSA7 (0.017912)
29. feature Xp-6d (0.017212)
30. feature Xp-5d (0.015521)
31. feature MID (0.015481)
32. feature Xp-4d (0.015280)
33. feature ETA_beta_ns (0.015276)
34. feature ABC (0.015259)
35. feature SlogP_VSA6 (0.014436)
36. feature ETA_eta_RL (0.014033)
37. feature nBondsM (0.014014)
38. feature Xp-1d (0.013771)
39. feature Zagreb2 (0.013283)
40. feature nC (0.010453)
41. feature MPC3 (0.009406)
42. feature nBondsKD (0.008465)
43. feature C3SP2 (0.008404)
44. feature Zagreb1 (0.008156)
45. feature NaasC (0.007751)
46. feature MPC2 (0.007087)
47. feature nAromAtom (0.007081)
48. feature NaaCH (0.006717)
49. feature nAromBond (0.006469)
50. feature nBondsA (0.006134)
51. feature MWC01 (0.005902)
52. feature nRing (0.005875)
53. feature nBondsO (0.005863)
54. feature naRing (0.003981)
55. feature n6Ring (0.002921)
56. feature n6aRing (0.002301)

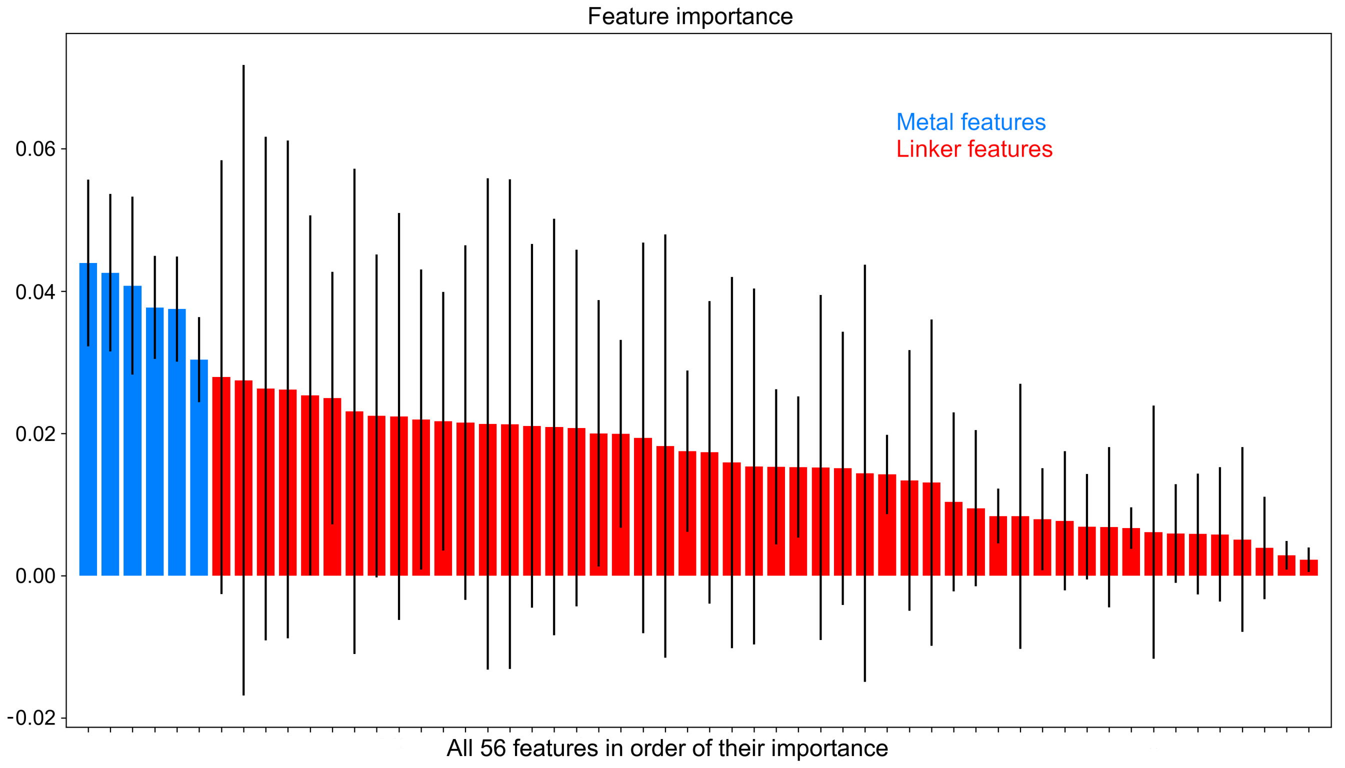

Figure S5. Feature importance for model 1 shown in order of their importance together with their standard deviation shown as the black vertical lines. Metal features are shown as blue bars and linker features are shown as red bars.

Figure S5 shows the 56 features listed above together with their standard deviation. All six features describing the metal (shown as the blue bars) are identified on average as having higher importance and smaller inter-tree standard deviation (shown as the black vertical lines) compared to the linker features (shown as the red bars). This suggests that the six metal features are consistently identified as important, whereas for the 50 most relevant features of a chemically diverse pool of linkers, their importance varies significantly between different trees. This is consistent with our ML model capturing multiple mechanisms that control porosity in a MOF through the choice of linkers.

2.4 Using the Jupyter notebook

Both the 1M1L3D dataset and the ML models we built are freely available to download as a Jupyter notebook and can be either used as is for making predictions for a user-defined metal-linker combination or adapted for different use. The notebook includes an interactive principal component analysis (PCA) map of the entire 1M1L3D dataset that allows the user to readily explore the domain of applicability of the tool and quickly identify reported MOF structures that are chemically similar to the given metal-linker combination (or indeed to see if this combination has been previously reported to produce a MOF). The values of the cut-off PLDs and any parameters of ML models can be easily changed by the user. Both the decomposition and the ML procedures are seamlessly applicable to 1D and 2D MOF networks and can be extended to predict other material properties of interest.

To demonstrate how the Jupyter notebook can be used to predict guest-accessibility of the MOF produced by a user-defined metal-linker combination that the model has not seen before, we selected a CSD entry HOYRAQ[14] with a PLD of 3.82 Å that was not part of the training set (it was part of the validation set). The section of the notebook that allows users to test their own combinations is in the second half and identifiable by the section title: “Use the models for new predictions”.

After entering the SMILES code for the linker and the metal identity:

linker = 'OC(=O)c1cc(Nc2cncnc2)cc(c1)C(=O)O'

metal = 'Cu'

The sequence of models correctly predicts that the expected PLD should be within the range 2.4 Å < PLD < 4.4

Moreover, in Figure S6, the target composition just tested is positioned (red dot) in a principal component map of the entire 1M1L3D dataset. In the notebook provided, this map is interactive, and it is possible to zoom in, to identify the most similar compositions which are the closest from the target. Additionally, each point corresponding to a CSD entry has been coloured according to the Tanimoto coefficient that quantifies chemical similarity between the target linker and the linker from the dataset. This map is intended as an illustration of the domain of applicability of the ML models developed in this work but can also be used to search for potential synthetic protocol of a target system by looking at MOF of similar composition.

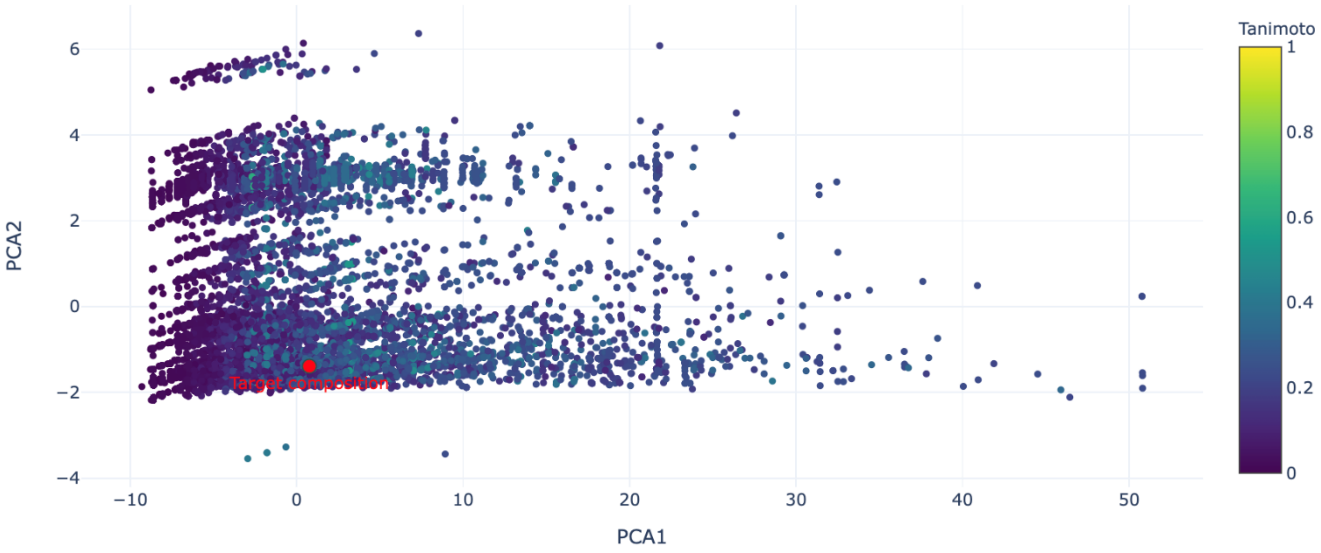

Figure S6. A screenshot of the interactive principal component map from the Jupyter notebook that shows the position of user-defined metal-linker combination as the red dot. Each point corresponds to a CSD entry from the 1M1L3D dataset coloured according to the Tanimoto coefficient that quantifies chemical similarity between the target linker and the linker from the dataset.

In addition, in the main text we mentioned that the PLD thresholds defined for the series of models trained in this work could be easily modified according to users need. It can be done from the start, in the cell entitled “Thresholds”.

Access to Jupyter notebook and the 1M1L3D dataset files

The Jupyter notebook and the 1M1L3D dataset files are freely available under this permanent URL referenced by DOI:

https://dx.doi.org/10.17638/datacat.liverpool.ac.uk/1494

Please cite the corresponding paper if you used the 1M1L3D dataset or these machine learning models in your work.

References

[1] T. F. Willems, C. H. Rycroft, M. Kazi, J. C. Meza, M. Haranczyk, *Microporous Mesoporous Mater.* **2012**, *149*, 134–141.
[2] P. Z. Moghadam, A. Li, X. W. Liu, R. Bueno-Perez, S. D. Wang, S. B. Wiggin, P. A. Wood, D. Fairen-Jimenez, *Chem. Sci.* **2020**, *11*, 8373–8387.
[3] P. Z. Moghadam, A. Li, S. B. Wiggin, A. Tao, A. G. P. Maloney, P. A. Wood, S. C. Ward, D. Fairen-Jimenez, *Chem. Mater.* **2017**, *29*, 2618–2625.
[4] N. M. O'Boyle, *J. Cheminform.* **2012**, *4*, 1–14.
[5] N. M. O. Boyle, M. Banck, C. A. James, C. Morley, T. Vandermeersch, G. R. Hutchison, *J. Cheminform.* **2011**, *33*, 1–14.
[6] D. Weininger, *J. Chem. Inf. Comput. Sci.* **1988**, *28*, 31–36.
[7] G. A. Landrum, “RDKit: Open-source cheminformatics,” **2006**.
[8] T. A. Halgren, *J. Comput. Chem.* **1996**, *17*, 490–519.
[9] A. Mauri, V. Consonni, M. Pavan, R. Todeschini, *MATCH* **2013**, *56*, 1-237–248.
[10] H. Moriwaki, Y. S. Tian, N. Kawashita, T. Takagi, *J. Cheminform.* **2018**, *10*, 4.
[11] N. M. O. Boyle, M. Banck, C. A. James, C. Morley, T. Vandermeersch, G. R. Hutchison, *J. Cheminform.* **2011**, *3*, 33.
[12] J. Gasteiger, M. Marsili, *Tetrahedron* **1980**, *36*, 3219–3228.
[13] F. Pedregosa, G. Varoquaux, A. Gramfort, V. Michel, B. Thirion, O. Grisel, M. Blondel, P. Prettenhofer, R. Weiss, V. Dubourg, J. Vanderplas, A. Passos, D. Cournapeau, M. Brucher, M. Perrot, E. Duchesnay, *J. Mach. Learn. Res.* **2011**, *12*, 2825–2830.
[14] Z. Chen, K. Adil, Ł. J. Weseliński, Y. Belmabkhout, M. Eddaoudi, *J. Mater. Chem. A* **2015**, *3*, 6276–6281.
